# Web-based searching for abortion information during health emergencies: a case study of Brazil during the 2015/2016 Zika outbreak

**DOI:** 10.1080/26410397.2021.1883804

**Published:** 2021-02-18

**Authors:** Tiziana Leone, Ernestina Coast, Sonia Correa, Clare Wenham

**Affiliations:** a Associate Professor of Health and International Development, Department of International Development, London School of Economics, London, UK; b Professor of Health and International Development, Department of International Development, London School of Economics, London, UK; c Co-Chair Sexuality and Policy Watch/Observatório de Sexualidade e Política – ABIA, Rio de Janeiro, Brazil; d Assistant Professor of Global Health Policy, Department of Health Policy, London School of Economics, London, UK

**Keywords:** abortion, Google Trends, Zika, microcephaly, tele-health, internet

## Abstract

Sexual and reproductive health needs and access are often neglected during health emergencies. The 2015/2016 Zika epidemic is an example of priorities shifting to the detriment of women’s health needs. The internet is a key tool for abortion knowledge sharing and seeking in countries where abortion is not legally available and it is also a key resource for tele-health. Yet, we know very little about how people use the internet, and the type of information searched for, to access abortion information and services. The aim of this study is to analyse to what extent and how the internet was used as a resource for abortion information during the Zika outbreak and its aftermath in Brazil in 2015/2016. Using Google Trends and Analytics data, we analyse contextually-specific abortion searches using standardised terms that reflect the overall representation of searches at that time alongside weekly levels of Zika incidence. The results show a heightened use of combined search terms for abortion and Zika, as well as abortion and microcephaly, suggesting a rise in abortion information searching linked to the epidemic. These searches were highly correlated with the level of Zika incidence. This study confirms the use of the internet for information seeking during a public health emergency. It demonstrates the need for appropriate internet resources to improve access to abortion information, especially in countries where abortion is highly restricted and stigmatised.

## Résumé

Les besoins de santé sexuelle et reproductive et l’accès aux services sont souvent négligés pendant les urgences sanitaires. L’épidémie de maladie à virus Zika en 2015/2016 est un exemple de réorientation des priorités au détriment des besoins de santé des femmes. Internet est un outil clé pour le partage et la recherche de connaissances sur l’avortement dans les pays où l’interruption de grossesse n’est pas légale et c’est aussi une ressources majeure pour la télémédecine. Pourtant, nous savons très peu de chose sur la manière dont les gens utilisent Internet pour avoir accès aux informations et services relatifs à l’avortement, et le type de renseignement qu’ils recherchent. L’objectif de cette étude est d’analyser dans quelle mesure et comment Internet a servi de ressource pour les informations sur l’avortement pendant et après l’épidémie de maladie à virus Zika au Brésil en 2015/2016. Au moyen des données de Google Trends et Google Analytics, nous avons examiné des recherches contextuelles sur l’avortement en utilisant des termes types qui reflètent la représentation globale des recherches à cette période, parallèlement aux niveaux hebdomadaires d’incidence de la maladie à virus Zika. Les résultats montrent une utilisation accrue de termes de recherche associés pour l’avortement et Zika, de même que pour l’avortement et la microcéphalie, ce qui semble indiquer une hausse de la recherche d’informations sur l’avortement liée à l’épidémie. Ces recherches avaient une forte corrélation avec le niveau d’incidence de la maladie à virus Zika. L’étude confirme l’utilisation d’Internet pour la recherche d’informations pendant une urgence de santé publique. Elle démontre la nécessité de disposer de ressources appropriées sur Internet pour élargir l’accès aux connaissances sur l’avortement, spécialement dans les pays où l’interruption de grossesse est extrêmement restreinte et stigmatisée.

## Resumen

Las necesidades de salud sexual y reproductiva (SSR) y el acceso a los servicios de SSR a menudo son olvidados durante emergencias de salud. La epidemia de Zika en 2015/16 es un ejemplo de cuando las prioridades cambian en detrimento de las necesidades de salud de las mujeres. Internet es una herramienta clave para el intercambio y la búsqueda de conocimientos sobre aborto en países donde el aborto no está disponible legalmente; además, es un recurso clave para la telesalud. Sin embargo, sabemos muy poco sobre cómo las personas utilizan internet, y sobre el tipo de información buscada, para acceder a información y servicios de aborto. El objetivo de este estudio es analizar en qué medida y cómo se utilizó internet como recurso para obtener información sobre el aborto durante el brote de Zika y posteriormente en Brasil en 2015/16. Utilizando *Google Trends* y *Analytics data*, analizamos búsquedas sobre aborto en contextos específicos, empleando términos estandarizados que reflejan la representación general de búsquedas en ese tiempo junto a niveles semanales de incidencia de Zika. Los resultados muestran mayor uso de los términos de aborto y Zika combinados durante las búsquedas, así como de aborto y microcefalia, lo cual indica un aumento en la búsqueda de información sobre aborto asociado con la epidemia. Estas búsquedas estaban muy correlacionadas con el nivel de incidencia de Zika. Este estudio confirma el uso de internet para la búsqueda de información durante una emergencia de salud pública. Demuestra la necesidad de tener recursos de internet adecuados para mejorar el acceso a la información sobre aborto, especialmente en países donde el aborto es muy restringido y estigmatizado.

## Background

Health emergencies, including Ebola, Zika and COVID-19, are characterised by neglect of gendered differences in experiences of, and responses to, infectious disease outbreaks. Sexual and reproductive health (SRH) has been highlighted as a critical health issue that has been ignored during health emergencies.^1^ This neglect has multiple sources: shifts of resources (human and financial) to focus on the health emergency; disruptions to commodity supply chains; and deliberate moves to erode existing rights at a time when attention is focused on the emergency and the provision of essential services. Such trends have been evident in all recent health emergencies: Zika in Latin America,^2–5^ Ebola in Sub-Saharan Africa^6,7^ and COVID-19 worldwide.^8–10^

Previous research has highlighted the need for the response to infectious disease outbreaks to include SRH as a key component to make sure women’s health and rights are protected.^5^ A critical component of SRH in health emergencies is access to abortion, and abortion-related information and services, but we know little about how people navigate to find information about abortion during health emergencies when there is a double “tyranny of the urgent”, for both unwanted pregnancies and the health emergency.^1^

The knowledge environment is a critical component of trajectories to abortion-related care and includes both generalised discourses around abortion and the specific information that an individual might (not) know or seek.^11^ The content and quality of information provided or found can be correct or incorrect, directive or non-directive, relate to a range of issues (safety, availability, legality, cost), come from a range of sources (national or international) and use different technologies and media. Although the impact of internet access on abortion-seeking behaviours is unclear, previous evidence from the USA has shown that accessing the internet for abortion-related information is directly related to the level of legal restrictions on abortion.^12^ Where restrictions are higher, a higher volume of internet searches for abortion-related terms is found. A need to seek information secretly because of abortion stigma and/or illegality adds additional barriers to information and care-seeking for abortion.^13,14^

Reflecting increasing global internet access, the use of the internet to search for information on abortion, especially in contexts where abortion is legally restricted, is growing.^12^ Tele-health, e-health and telemedicine (the use of the internet and/or phone services) to support medical abortion (the use of mifepristone and misoprostol to terminate pregnancy) are a key feature of the abortion access landscape and can provide access to information and care in a range of legal settings.^15,16^ Tele-health is one strategy deployed as a response to a health emergency, such as in the UK during COVID-19.^17^ Immediately after the declaration of the Zika epidemic as a Public Health Emergency of International Concern (PHEIC), requests for services from Women on Web, a tele-health platform which provides medical abortion in restricted settings, increased in Brazil by between 36% and 108%.^2^ Demand for existing e-health information and services may also increase in routine times of non-crisis.^18^

In addition, there has been a growing presence on the web of feminist and reproductive rights groups who have exploited digital media to get messages across. Little is known, however, about how people search for abortion-related information online: what sort of language is used, and how the content and volume of searches change during health emergencies. Most of the evidence to date is based on data from tele-health organisations; these data are organisation-specific and reflect the market capture of a subset of people. An analysis of the background of Brazilian women accessing the services of the Women on Web website showed that almost half of the people contacting the service are poor and of younger age (below 25 years old),^19^ while women with internet access are usually more educated and wealthier compared to the general population.^20^ With the rise of tele-health, if internet-based information relating to abortion is to be better designed to meet people’s informational needs, we first need to understand what those information needs are, especially in contexts where abortion is legally restricted.

In this paper, we investigate if and how the internet was used for seeking abortion information during the 2015/2016 Zika health emergency in Brazil. The Zika epidemic in Latin America emerged in 2015 and the birth of children with congenital Zika syndrome (including microcephaly), brought to the fore discussions about reproductive rights during health emergencies, as women became fearful about their current or future pregnancies.^3^ We analysed two sets of internet-based data. First, we analysed Google Trends (GT) data related to abortion key words, before, during and after the Zika crisis, to explore whether Zika had an impact on web-based searches. We then analysed the volume of traffic from Brazil to a tele-health provider, Women Help Women, using Google Analytics (GA).

This study contributes evidence about how people search for abortion-related information during health emergencies.^11^ Analysing behaviours through the lens of internet interest sheds light on the type of information needed at the time of a health crisis. The information we have compiled for this study is of interest both for the impact that Zika had on abortion information-seeking, and also to improve information available to people seeking to terminate a pregnancy and to understand any changes in demand for abortion during health emergencies.

## Context

Brazil was the country most affected by the Zika outbreak, and has one of the most restrictive abortion legislations in Latin America, permitting termination to save a woman’s life, in cases of rape and incest or if a foetus has anencephaly (where it develops without a brain).^21^ Calls for abortion decriminalisation were sparked off in the 1980s in the early days of democratisation and increased until 2005, when a full abortion law reform, tabled by the Executive Branch, was impeded by the first corruption crisis of the Worker’s Party (PT) government.^22^ Since then, the struggle for abortion rights has, by and large, lost ground, except for a few key moments.

In 2012, the Supreme Court granted the right to abortion in the case of anencephaly.^23^ Then, in late 2014, the problem of unsafe abortions once again reached the press after two women died of botched illegal abortions in Rio. Feminists stepped up their campaign for change and a year later, in October and November 2015, took to the streets to protest against imminent threats to the legality of abortion in the case of rape. These demonstrations coincided with the eruption of the Zika crisis in relation to pregnancy. The sequence of events reactivated the public debate on abortion rights, leading to a Senate debate on legalisation and the tabling of two lawsuits at the Supreme Court. The first debate, presented in 2016, called for the social and individual rights of women affected by Zika and was dismissed in 2020. The second, presented in March 2017, called for the legalisation of abortion upon demand until the 13th week of pregnancy. A decision is still pending.^24^

In Brazil, access to the internet within households is high, but unequal across the country, reflecting socio-economic development inequalities across regions (Table 1). The south and centre-west states are the most economically prosperous and include states such as São Paulo, Rio de Janeiro and Minais Gerais. These states have higher access to the internet while the northeast states, a region which includes states such as Bahia and Pernambuco, have the lowest indicators.

**Table 1.** Background data Brazil 2015–2016

	Households using the internet (% of population) 2016^a^	GRP per capita in USD^b^	Microcephaly^c^ Total number of cases Jan 2015/Nov 2016	Zika cases in 2016 (/100,000 inhabitants)^d^	Abortion rate (per 1000 women aged 15–49)	Abortion legal status^e^
Total	69.4	**8,727**	1950	105.3	32	Allowed to save woman’s life and in case of rape
North	62.4	5,430	71	74.2		
North-East	56.5	4,495	1487	134.4		
South East	76.7	11,294	270	106.2		
South	71.3	10,379	23	3.4		
Centre-West	74.6	11,119	88	222		

^a^ https://www.ibge.gov.br/estatisticas/multidominio/ciencia-tecnologia-e-inovacao/17270-pnad-continua.html?=&t=resultados.

^b^ Source: https://agenciadenoticias.ibge.gov.br/agencia-sala-de-imprensa/2013-agencia-de-noticias/releases/23038-contas-regionais-2016-entre-as-27-unidades-da-federacao-somente-roraima-teve-crescimento-do-pib.

^c^ https://www.thelancet.com/journals/lancet/article/PIIS0140-6736(17)31368-5/fulltext.

^d^ https://www.saude.gov.br/images/pdf/2017/fevereiro/05/2017_002-Dengue%20SE52_corrigido.pdf.

^e^ https://reproductiverights.org/worldabortionlaws accessed 14/04/2020.

In countries like Brazil where the use of social media is historically high and access to abortion is limited, the search for information on pregnancy termination and medical abortion has expanded geometrically in the last two decades. This continued even when, in 2006, severe restrictions were imposed on internet advertising on abortion by ANVISA (Agência Nacional de Vigilância Sanitária), the national regulatory health agency.^25^ Access to medical abortion through tele-health in Brazil has been previously researched.^19^ Despite the legal restrictions on abortion, telemedicine was accessible for several years until the Government restricted access to misoprostol by attempting to control the flow of information about misoprostol on the internet.^16^

**Figure 1.**
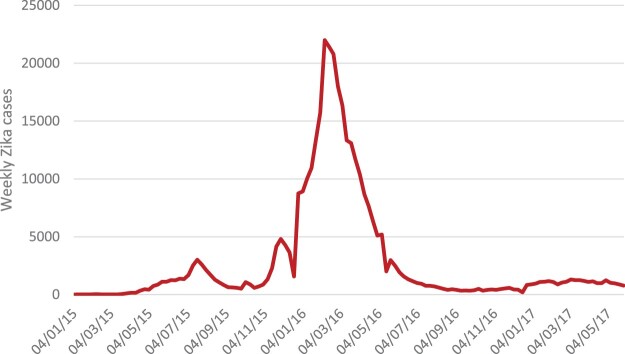
Zika incidence cases Brazil 1/1/2015–31/5/2017. Source: PAHO weekly data https://www.paho.org/data/index.php/en/mnu-topics/zika/524-zika-weekly-en.html

The Zika epidemic showed worsening trends in May 2015, with a clear peak in February 2016, progressively slowing down by September 2016 (Figure 1). National-level data on Zika in Brazil obscure significant state-level variation (Table 1). The beginning of the epidemic was mostly in the northeast and north, with Pernambuco in the northeast reporting the highest percentage of suspected and confirmed cases of microcephaly, one of the symptoms later referred to as congenital Zika syndrome (CZS), with 231,725 suspected and 137,288 confirmed cases throughout Brazil. By the end of the epidemic the highest Zika incidence was reported in the southeast followed by the northeast, while the highest incidence of microcephaly was reported in the northeast.

To situate and understand internet searches related to abortion during the Zika outbreak it is important to understand the timeline of events in Brazil. Zika was first reported in Brazil in May 2015 with reports from health clinics of an unknown rash and fever.^26^ However, it was not until later in 2015 that a higher than expected incidence of microcephaly began to emerge. In northeast Brazil, by November 2015, several hundred cases of microcephaly were being reported each month.^27^ Associations began to be made between the mysterious rash in mothers and children born with microcephaly,^28^ leading to multiple investigations and declarations of emergencies within state governance structures to facilitate emergency funding for investigations and preventive vector control. In November 2015, the Ministry of Health declared a national public health emergency and confirmed the link between Zika and microcephaly.^29^ In the midst of the emergency the Brazilian government released a statement asking women to postpone getting pregnant, a statement which was subsequently widely criticised.^30^ During this period, the epidemic received wide media coverage.^31,32^ These emergency declarations facilitated greater financing for state governments in their response efforts and justified the use of the military to support community health workers and vector control agents to combat mosquitoes.^29^ By February 2016, WHO had declared Zika and microcephaly a Public Health Emergency of International Concern.^33^ In March 2016, an article in the New England Journal of Medicine consolidated the link between microcephaly and Zika.^34^ The end of the public health emergency was declared in November 2016, and the Brazilian government ended the state of emergency on the 11th of May 2017. A series of analyses that have been conducted subsequently on the media coverage of the epidemic also showed that the peak occurred in February 2016. This coincided with the WHO’s declaration of the international emergency. Media interest started gradually in November 2015, finally disappearing after May 2016.^31,32^

Why northeast Brazil emerged as the epicentre of the CZS outbreak has yet to be fully explained, but possibilities include co-infection with dengue fever,^35^ increased severity of Zika infections,^36^ lower access to contraceptives, leading to a higher rate of sexual transmissions,^37^ lower access to water and sanitation facilities, lower nutritional status of mothers and the lower socio-economic background of the region (meaning less access to insecticides/housing with screens, running water etc.). Fake conspiracy narratives also erupted, asserting that states where modified mosquitoes, which are unable to transmit arboviruses, had been introduced as a form of vector control, showed greater increases in CZS as a result. This caused widespread panic which detracted from public health efforts.^38^

As noted above, the epidemic reopened the media debate on abortion rights, with many feminist and other pro-abortion rights voices calling, once again, for liberalisation of the law.^39^ While it is not clear how the public reacted to these calls, the hypothesis may be raised that, under the impact of this intensified public debate, women may have felt more comfortable searching for information on, and accessing abortion pills, online. What was clear at the time, however, was that the Minister of Health had asked women to avoid pregnancy without creating a facilitative environment in which to do so – i.e. one where women have autonomy to decide their reproductive futures.^5^ Regulatory and structural barriers remained for women seeking to access contraception, emergency contraception and abortion.

## Data and methods

In this study we analysed two sources of web-based data: GT data; and GA data from a web-based tele-health provider of Medical Abortion (MA).

### Google Trends

Data was accessed on the 14 April 2020. We used GT^40,41^ weekly data between the 1st of January 2015 and the 31st of May 2017, a timeframe which spans the period before, during and after the peak of the Zika epidemic.^42^ We did this to track trends in searches about abortion which are useful not only for their overall interest, but also to track fluctuations across time in line with other policy changes during this timeframe. We normalised the GT data by total searches, an established technique in internet studies,^43–46^ meaning that:

$$\begin{array}{rl} search\;interest & =(\#;\;of\;queries\;for\;keyword)/ \end{array}(total\;GT\;queries)$$

Thus, our abortion-specific data trends are relative to the overall number of searches at that time in that location.^46^ This approach accounts for individuals doing the same search more than once within a short time period (Google does not specify the length of time), so each search is only counted once. Therefore, any deviation from the overall level of searches for terms associated with abortion would be significant and might be linked to an event or to an increase in media attention.

Searches were conducted in Portuguese and English using context-specific language identified based on key informant perspectives combined with insights from social media (Twitter) identified by the authors (Supplemental data, Appendix 1).

GT search results are proportionate to the time and location of a query using a systematic process:

- Each data point is divided by the total searches of the country/region and time range it represents to compare relative popularity. If this relative popularity is not established, then locations with the largest search volume would always be ranked highest.
- The resulting numbers are then scaled on a range of 0–100 based on a topic’s proportion to all searches on all topics.

GT only shows data for popular terms and search terms with low volume appear as “0”. If over the period analysed there are not enough searches to create a trend, GT would not produce any data. The analyses exclude queries with special characters, such as apostrophes, because these are filtered out by GT. Different regions that show the same search interest for a term do not always have the same total search volumes.

There are established approaches to correct for shifting baselines in overall internet search volume. We scaled relative monthly search volume for each keyword by dividing it by a benchmark term. We selected benchmark terms – *software*, *computer*, *life*, *love* – based on established approaches.^44^ The four benchmark terms were selected to represent a range of higher/lower relevance for the focus of our study – abortion – to assess the sensitivity of this correction to the choice of benchmark term. Prior research has established that these four terms are constant in popularity and unaffected by changing volumes of internet searches. We checked the trend over the period and confirmed them to be constant. For presentation, we only show the results relative to one benchmark term (love); results for the other three benchmark terms were similar. Our use of this approach allows us to account for the fact that our analyses can only consider search frequency, and not the final landing webpage of the search.

Finally, we considered comparisons across states; we added this intra-national analysis to explore whether there was any relationship between internet searches and Zika incidence, which varied geographically within Brazil. GT reports in which location the search term was most popular during the set time frame. The results are relative with values on a scale from 0 to 100, where 100 is the location with the most popularity as a fraction of total searches in that location.^40,41^ The results are a relative measure of the popularity of a term, not absolute, and are not weighted by size of the state.

We conducted two sets of GT analyses. First, we applied Mann-Kendall tests to detect any trends over time.^47^ This non-parametric test is usually used for time series data which are not normally distributed. Mann-Kendall tests the hypothesis that the data has no trend by producing a coefficient – *tau* – which observes the trends of each of the search terms we considered across time. Second, we looked at correlations between the key search terms to understand whether there is a common trend between them, for example, searches on abortion and Zika. For this we used the Spearman *rho* test which is a test between two non-normally distributed trends.^44,47,48^

### Google Analytics

We analysed the GA data for the tele-health medical abortion provider Women Help Women. We focused on one tele-health provider only as it has a large market capture in Brazil, and we have pre-established relationships and so were able to access their data. GA is also being used as a comparative narrative to GT which is the main focus of our analysis. Other studies have already analysed GA data on another large provider, Women on Web.^2^ GA is usually used as a marketing tool to track the traffic on website pages. Analytics measures the number of “clicks” that land on website pages during the same period we observed for GT.^49^ Information can be retrieved by date, place of location of the search, time spent on the page, whether the individual typed the page address directly or arrived through other websites (e.g. Google) and internet service provider. It can tell us how many visits a day a specific page had and how long on average users stayed on the page as well as repeated visits. Overall, the analysis of GA for an e-health website can give us an idea of trends in interest in medical abortion over a time period. We looked at the number of visits on the main Women Help Women website for the period 1 November 2015–31 May 2017. For the analysis of these data we used the same statistical tools used for GT data. We calculated the monotony of the trend data with the Mann-Kendal *tau* and the Spearman *rho* to test the correlation of the trend with Zika incidence levels. No ethical clearance was sought as we analysed secondary anonymised data.

## Limitations

The limitations of our data and analyses are common to research that uses internet search data. First, there are substantial inter- and intra-country variations in internet availability, accessibility and use. Second, we do not know anything about the characteristics of the person conducting the search. In every setting, individuals with greater resources (education, financial, computer/phone ownership, etc.) have lower barriers to accessing internet-based information.^45^ There are limitations to the GT algorithm because it relies on the relative traffic of search during the period. Therefore, if a popular topic accounts for more searches during the same period, GT is unable to capture significant changes in patterns. Finally, it would have been analytically insightful to compare search term trends with microcephaly incidence data as well. Consistent and reliable weekly data on microcephaly were not available; consistent weekly data were only available for Zika incidence. But we consider Zika values to represent an accurate picture of the intensity of the emergency and possibly of greater resonance at the time. In addition, an analysis of the incomplete microcephaly data showed a high level of correlation with Zika epidemiological trends.

GA data has many similar shortcomings to GT, including not being able to know the individual’s characteristics and, importantly, what they do with the information obtained from the website. In addition, it is often not possible to get the level of regional detail (we only get state level of information) necessary to have an in-depth analysis of the location of the searches.

## Results

**Figure 2.**
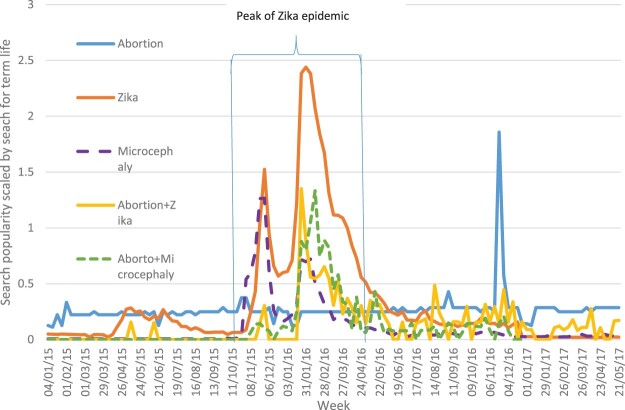
Google Trends selected search terms Brazil 1/1/15–31/5/17

We present the results of the Portuguese searches only, as the English searches either did not produce enough data to display or did not report any significant trend. We show only trends where data were available and report the most significant trends (e.g. there were enough data and the trend showed a meaningful pattern). The trends show a clear prominence of searches including the word Zika (Figure 2) around the time of the peak of the epidemic (November 2015–April 2016). This is probably a reflection of increased media attention after the Brazilian government and the WHO declarations of a public health emergency. The peak of the searches also clearly matches the peak of the epidemic (Figure 1).

The relative volume of searches that only included the word *aborto* (abortion) is consistently low across the same time period. However, a clear increase is seen around the time (November/December 2016) of a national debate about the legal status of abortion in Brazil when the Brazilian Supreme Court issued an opinion on legalisation of abortion when judging a criminal case under which several abortion providers where arrested.^39,50^

On its own, searches related to the word *aborto* show no relationship with the Zika health emergency and there are no trends for searches related to medical abortion (misoprostol) or abortion pills (*comprimido*, misoprostol, *pastilha* or *medicamento*). Abortion and legalisation words together showed a spike again in the summer of 2016 at the time of the Supreme Court opinion on abortion legalisation.

What is noticeable is the increase in searches that combine *aborto* and Zika during the peak of the Zika outbreak in Brazil. Those who were searching for abortion-related information were clearly looking in conjunction with Zika.

Regional variations in search term volume and trends are mixed (Supplemental data, Table A1). More economically developed states (e.g. Rio de Janeiro, Minas Gerais) show higher volumes of searches. *Zika*, *aborto* and *microcefalia* (microcephaly) seem to reflect interest in all the Brazilian states in terms of popular searches. However, the combinations of search terms (e.g. *Zika + aborto* or *aborto + microcefalia*) are popular only in a smaller number of states and often do not report enough data to show any values for those terms. During the period of observation, the two regions in Brazil with the highest searches on Zika and abortion (*Zika + aborto*) together were the State of Alagoas and the state of Rio de Janeiro, followed by the state of Bahia. While Rio de Janeiro and Bahia were the states with high incidence of Zika in the country during the study period, they were not states with a high prevalence of either Zika and/or microcephaly.

The analysis of the GA data from the tele-health abortion provider Women Help Women (WHW) showed a different trend altogether. With these data we wanted to understand whether the epidemic sparked a rise in seeking medical abortion services. The data for WHW showed an increase in interest during this period (landings on the WHW page more than tripled between January 2015 and December 2016). However, this interest continued to rise until the end of the observation period, showing no real link with the epidemic (e.g.: there was no decline after the end of the peak period). The *tau* value of the trend is significantly positive (*tau* = 0.640, *p* < 0.001) but the correlation with the Zika incidence is not significant.

### Trend analysis

Only one of our search terms (*aborto + comprimido*) showed a monotonic trend (Table 2), meaning that where increases in search term volume occurred, they were temporary. This set of analyses gave us a general check that the level of interest was therefore confined to the epidemic and not to a general trend. *Zika*, as expected, was increasing as well as the combined *aborto + Zika* search. Although weak (−0.046), the trend of the term *aborto* seemed to be declining over the period. More interestingly, *aborto + comprimido* was significantly increasing over this period (0.085, 0.001 < *p* < 0.05) showing a possible increase in interest on medical abortion. However, the same was not true for the trends of the search of the word misoprostol which could be as likely to be used for medical abortion as *comprimido*. The *tau* value for the period was 0.639 (*p* < 0.000), showing a significantly strong increasing trend in line with the analysis of the data we reported in the previous section.

**Table 2.** Mann-Kendall trend test tau values for GT trends

Search term	Brazil
Abortion	−0.046
Zika	0.058
Misoprostol	−0.097
Abortion + Zika	0.030
Abortion + pill	0.085**
Microcephaly	0.010
Abortion + microcephaly	0.030
Abortion + misoprostol	−0.021

Significance: **p < 0.05.

The *tau* value should be interpreted as a correlation coefficient between −1 and +1. A negative number shows a decline over time. A positive number shows an increase over time.

Where the correlation between the search terms is significant (Table 3) it means that the terms were either increasing in popularity (positive) at the same time or that they were diverging in popularity (negative). Searches for combined *Zika + aborto* terms are positively correlated with both the searches of the terms *aborto* (0.286, *p* < 0.001) and Zika (0.575, *p* < 0.001). While Zika and *aborto* are not correlated, the terms *microcefalia + aborto* are correlated, perhaps showing increased concern for a potential outcome of the disease rather than the disease itself. Search terms about legal or legalisation (e.g. *aborto *+ *legal*) were not significantly associated with either trends or other search terms at the time.

**Table 3.** Correlation terms Spearman rho correlation, GT search terms and Zika incidence Brazil 1/1/15–31/5/17

	Zika	Abortion	Microcephaly	Misoprostol	Abortion+Zika	Abortion+Microcephaly	Abortion+pill	Zika Incidence
Zika	1							
Abortion	0.0218	1						
Microcephaly	0.696***	0.323***	1					
Misoprostol	0.053	0.091	0.246**	1				
Abortion+zika	0.282***	0.067	0.287***	0.187	1			
Abortion+microcephaly	0.708***	0.249*	0.755	0.226*	0.437***	1		
Abortion + pill	0.029	0.305**	0.115	−0.171	−0.138	0.069	1	
Zika incidence	0.822***	−0.117	0.517*	−0.069	0.054	0.434*	−0.002	1

***p < 0.001, **0.005 < *p* < 0.001, *p > 0.10.

The incidence shows a strong correlation with the terms Zika and microcephaly, as expected, but also with the combination *aborto + microcefalia + Zika* (0.434, *p* < 0.001). Misoprostol is positively correlated with the search *microcefalia* (0.246, 0.01 < *p* < 0.05) and *aborto *+* microcefalia* (0.226, 0.01 < *p* < 0.05).

No significant correlation was reported between the WHW GA data and the Zika incidence trends.

## Discussion and conclusions

This study has shown clearly that people were searching online for information about abortion in connection with Zika and/or microcephaly over the Zika health emergency period. Most notably the study highlighted discrepancies between the searches and the epidemic across states. This could be because of state-level differences and, in particular, between the northeast and south of the country,^5,26,32^ reflecting a combination of factors which include level of internet access, perception of risk of microcephaly and other differences across the states consistent with other studies.^12^ Overall, there is a mismatch of gravity of the epidemic and surge of internet search at state level. Most likely this is due to availability of access to internet, educational levels and awareness of availability of services/information online. This is in contrast with previous research in the USA that showed an inverse relation between access to internet for abortion services and level of legalisation within the state.^12^

Tele-health and medical abortion also increased during the time of the epidemic, beyond what could be considered routine service provider growth. First, the rise could reflect the increase in visibility of WHW on the web (they were set up in 2014 just before the epidemic started). Second, when comparing with the results by Aiken et al., Women on Web conducted a specific campaign on Zika and abortion in Brazil at a time when their presence was already strong.^2,19^ In addition, the increase in debate on the legalisation of abortion could have increased individuals’ knowledge of the availability of medical abortion services through the internet.^2,39^ Finally, the comparison would not be strictly appropriate as Aiken et al. used actual consultations in their study rather than GA, confined to a very limited period which included the peak of the epidemic only.^2^ Using these data, we are unable to investigate whether the search trends are as a direct consequence of the epidemic or driven by the wider debate on abortion legislation.^39^ We believe the findings demonstrate increasing awareness of these services in Brazil as a consequence of Zika. Nevertheless, we could speculate that given the increase in access to search words linking abortion and Zika, the epidemic was implicated in the upward trend in accessing e-health services, although we have no data on abortions to validate this.

In this study we can only make inferences about the reasons behind the trends. It could be that usually this kind of information is only available to more educated, wealthier individuals. We are also unable to account for how information is otherwise disseminated, such as through informal networks or social media groups such as on Facebook. More generally we lack information on the diffusion of information through informal networks, including word of mouth and gossip.^11^

Despite the data limitations, the analysis shows a clear need to investigate further the type of information individuals access at a time of health emergency, and how this affects their trajectory towards abortion (or not). This is particularly important in settings where information is restricted and access to services difficult.

Our analyses are the first to analyse GT data alongside the incidence of an epidemic and demonstrate the value of doing so. At the time of a heightened crisis because of an epidemic such as Zika or the current COVID-19 crisis, there needs to be a greater effort to provide quality information about how to access SRH services during changes to regular provision or the introduction of non-pharmaceutical interventions, such as lockdown. Very recent literature has highlighted how SRH issues get neglected in a time of pandemics and in times of public health crises in general.^8–10^ Prioritisation of COVID-19 interventions, for example, has come to the detriment of many other health services, with SRH and abortion services being among the most affected. Public health campaigns during a health emergency should include information on access to SRH services, which in turn should be ringfenced in resource reallocation. In a legally restricted setting, information on access to safe abortion must use effective channels. This study has demonstrated that the internet is one of those channels and we need to make sure that the information available is correct and accessible. Importantly, despite the current focus on COVID-19, Zika continues to circulate in Brazil. Indeed, the distortion of the health system for COVID-19 related care has led to a reduction of vector control, and thus we expect increased rates of arbovirus in the coming year(s).^51^ Future studies will need to examine fine-grained data to understand what kind of pathways individuals travel to access internet information about abortion. We need to better understand people’s informational needs, and their capabilities to meet those needs. Without such understanding, information and services cannot be designed to meet people’s needs, not only during health emergencies, but more generally.
